# Shifts in nutrient allocation in a gift-giving butterfly: a hidden consequence of water balance?

**DOI:** 10.1242/jeb.251506

**Published:** 2026-01-23

**Authors:** Chloé Chabaud, Natasha Tigreros

**Affiliations:** Department of Entomology, University of Arizona, Tucson, AZ 85719, USA

**Keywords:** Hydration, Resource allocation, Drought, Nuptial gift, Trade-offs, Stable isotopes, Metabolism, Reproduction

## Abstract

As climate change intensifies drought, understanding how animals maintain fitness under water stress is essential for predicting ecological resilience. Terrestrial animals use diverse behavioural and physiological strategies to avoid dehydration, yet the associated physiological and fitness costs remain poorly understood. Because water balance is tightly linked to nutrient acquisition and metabolism, mechanisms that enhance hydration may alter how animals allocate key macronutrients across vital functions. Here, we investigated how maintaining water balance – via increased water intake or reduced water loss – shapes nutrient allocation and trade-offs in the cabbage white butterfly (*Pieris rapae*), a species in which males transfer nutrient- and water-rich nuptial gifts to females during mating. Using controlled humidity treatments and stable-isotope tracing, we quantified how the hydric environment and mating status influence female allocation of nutrients – including nuptial gift-derived amino acids – to storage, fecundity and catabolism. We found that females in dry environments maintained water balance largely by acquiring nuptial gifts and by reducing respiratory water loss. However, dry conditions still altered nutrient allocation: females invested more lipids into eggs at the expense of long-term storage, and they reduced catabolism of an essential amino acid derived from the nuptial gift. These results show that mechanisms supporting water balance can indirectly reshape nutrient-use strategies, revealing physiological trade-offs that may influence longer-term fitness. More broadly, our findings highlight the tight coupling between water and nutrient economies and emphasize the need for a nutrient-explicit framework for understanding how animals cope with increasing aridity.

## Teaser

**Summary:** Water-balance strategies in a widespread butterfly can reshape nutrient allocation and expose physiological trade-offs, highlighting the interconnected nature of water and nutrient economies under increasing environmental aridity.

## INTRODUCTION

Water is an essential resource for all living organisms, comprising more than 60% of body mass in most terrestrial animals. Maintaining adequate water balance is critical to animal fitness, supporting key physiological functions such as thermoregulation, digestion and cellular homeostasis. However, the availability of free-standing water is often limited and increasingly variable as extreme weather events – such as droughts – intensify in some regions as a result of climate change (Cook et al., 2014; Trenberth et al., 2014). To avoid dehydration, animals can adjust their behaviour and physiology to increase water intake (e.g. through consumption of preformed water, i.e. water obtained directly from food rather than produced metabolically) and reduce water losses via cutaneous evaporation, excretion or respiration (e.g. Rozen-Rechels et al., 2019; Wharton, 1985). These water balance mechanisms have been extensively studied, particularly in arid-adapted species or in the context of climate change (Greenberg and Palen, 2021; Rozen-Rechels et al., 2019; Sinclair et al., 2024). Yet relatively little attention has been given to their potential costs or constraints, even though such mechanisms are often linked to processes of acquisition and metabolism of other nutrients. Understanding how organisms balance the benefits and costs of water balance mechanisms is critical for predicting whole-organism responses and resilience to changing environments (McCluney, 2017).

When animals obtain water through their diet, water balance becomes a key factor influencing their dietary preferences and foraging activity. Animals can regulate water intake by selectively feeding on foods with different preformed water and energy content (Clissold et al., 2014; Gedir et al., 2016). For example, nectar-feeding sunbirds can adjust their intake of diluted versus concentrated nectars to balance water and sugar acquisition, selectively feeding on nectars that match their physiological requirements (Köhler et al., 2012). When choosing among diets is not an option, animals can increase food intake until their water needs are met or, conversely, reduce food intake when consuming overly dilute foods leads to excess water intake (Köhler et al., 2012). A consequence of such a strategy, however, is that it can result in excessive intake of other food components and thus affect the optimal expression of other functions (Lee et al., 2008). While studies have typically focused on how organisms prioritize sources of caloric intake (e.g. protein versus carbohydrates), limitations in water availability may similarly lead to nutritional reallocation and trade-offs critical to overall fitness.

Nutritional shifts may also arise when the mechanism to maintain water balance involves changes in metabolic processes, such as metabolic water production. The production of metabolic water through the oxidation of dietary macromolecules is a widespread strategy for terrestrial animals to avoid dehydration (Bailey, 1923; Chown and Nicolson, 2004; Cockbain, 1961; Fuller et al., 2021). For example, birds and lizards under water stress are known to increase oxidation of proteins (e.g. Brusch et al., 2018; Gerson and Guglielmo, 2011; Rogers and Gerson, 2024), as protein catabolism produces more metabolic water than the oxidation of other macromolecules relative to the energy obtained, in part because it also releases bound water (Jenni and Jenni-Eiermann, 1998). Some invertebrate species rely more heavily on lipid or glycogen catabolism (e.g. Kalra and Gefen, 2012; Loveridge and Bursell, 1975; Schmidt-Nielsen, 1997). However, diverting lipids or protein for water production may reduce availability for other vital functions, such as reproduction, leading to physiological trade-offs. Thus, while their oxidation can provide a vital water source during dehydration, it comes at the cost of potentially impairing other aspects of fitness.

In addition to acquiring water metabolically, animals can conserve water by reducing their metabolic rate. In terrestrial environments, gas exchange through the respiratory system is a major route of water loss, as respiratory surfaces must remain moist to allow efficient diffusion of gases. Higher metabolic demands increase the rate and volume of gas exchange, thereby elevating evaporative water loss (Woods and Smith, 2010). Consequently, lowering metabolic rate can be an effective water-saving strategy. This adaptation has been documented in desert birds living across natural gradients of aridity (Tieleman et al., 2003), as well as in mammals (Nagy and Knight, 1994) and insects, which limit water loss via discontinuous gas exchange (Schimpf et al., 2012). While reducing metabolic rate helps conserve water, it can result in significant fitness costs. For instance, reproductive success may decline as a result of slower growth rates and reduced fecundity (Daan et al., 1990). Similarly, immune function can be compromised because of the reduced energetic investment associated with a lower metabolic rate (Rolff and Siva-Jothy, 2003).

Animals across a variety of taxa donate nutritive materials during courtship or mating, often referred to as nuptial gifts (Lewis and South, 2012). These gifts, typically provided by males to females, include prey items, regurgitated food and spermatophores (nutrient-rich packets of sperm) and can support female physiological condition and offspring production (Kamimura et al., 2021; Lewis and South, 2012). In insects, where the nutritional value of nuptial gifts is well established (Vahed, 1998), these materials have previously been proposed to serve a dual function in water-limited environments: providing essential nutrients for egg production and supplying water to mitigate female dehydration (Marshall, 1982). Subsequent research has supported their potential hydration benefits across insects. For example, water-deprived *Gryllodes sigillatus* crickets maintained offspring production comparable to that of their well-hydrated counterparts (Ivy et al., 1999), and female moths that mated with water-supplied males showed increased longevity, consistent with water transfer during copulation (Ryne et al., 2004). Behavioural studies further suggest that hydration opportunities influence reproductive decisions, as water-deprived *Callosobruchus maculatus* females mate more frequently (Edvardsson, 2007; Ursprung et al., 2009). Together, these studies highlight nuptial gifts as a valuable system to examine how water balance and nutrient allocation interact to shape fitness under environmental stress.

In this study, we tested the hypothesis that nuptial gifts in *Pieris rapae* butterflies help females cope with water stress while shifting the allocation of nutrients to other functions. *Pieris rapae* males transfer a nuptial gift (also known as a spermatophore) during mating. This gift is rich in sugars, nitrogen and water (Bissoondath and Wiklund, 1995; Boggs, 1981; Watanabe and Sato, 1996) and is known to substantially enhance female fecundity and longevity (Boggs and Gilbert, 1979). *Pieris rapae* butterflies’ wide geographic distribution (Ryan et al., 2019) exposes them to diverse humidity levels and frequently fluctuating water availability, making their potential for utilizing nuptial gifts for both water and nutrients particularly relevant. To understand how nuptial gifts contribute to maintaining water balance, we tested the impact of dry conditions on the hydration and fitness of mated versus virgin females. We then examined nutrient shifts and trade-offs using ^13^C-stable isotope to trace the allocation of lipids and a nuptial gift-derived essential amino acid to fecundity, catabolism and storage (Quinby et al., 2020). By integrating fitness and physiological measurements with real-time stable isotope analysis, our study provides a powerful approach to understand how terrestrial animals deal with water-limited environments.

## MATERIALS AND METHODS

### General rearing and ^13^C-labelled nuptial gifts

*Pieris rapae* (Linnaeus 1758) butterflies used for this experiment were from a laboratory colony that originated from a field population in UT, USA. Butterflies were reared from egg to 5 days old (early 2nd instar) on their host plant, *Brassica oleracea*. At this stage, larvae were transferred in pairs into 1 oz. (∼30 ml) plastic cups and fed *ad libitum* with a semi-synthetic diet (Morehouse and Rutowski, 2010; Tigreros, 2013). This diet minimized variation in the larval nutritional environment (e.g. maintaining 4.2% nitrogen by dry mass) and ensured optimal conditions for the development of males so they produce a protein-rich nuptial gift. Throughout their development, butterflies were kept in a walk-in environmental chamber on a 16 h:8 h light:dark photoperiod at 22°C and 50% relative humidity (RH).

To examine how females use nutrients from the male nuptial gift, we isotopically enriched the male larval diet. At 10 days old, when males can be reliably identified based on visible testes, individuals were switched randomly to either a ^13^C-leucine-labelled diet or a ‘control’ (unlabelled) diet until pupation (O'Brien et al., 2002). The ^13^C-leucine diet was made by the addition of 0.17 g l-leucine (1-^13^C, 99%, Cambridge Isotopes Laboratories, Inc.) per 0.5 l diet, and control diet was made with the same amount of l-leucine (non-labelled, 99%, Alfa Aesar). Manipulation of the male larval diet resulted in a clear isotopic distinction between control and labelled nuptial gifts transferred to females; the δ^13^C signature of labelled gifts was significantly higher than that of both the control gifts and the female diet (Fig. S1), confirming effective ^13^C enrichment. As essential amino acids cannot be synthesized *de novo* by adults, we hypothesized that they would be preferentially allocated to reproduction over metabolism, given their limited availability and importance for reproductive success.

To mate females with either ^13^C-labelled or control males, newly emerged females were transferred to a greenhouse (14 h:10 h light:dark cycle) and placed in a large cage (40×40×60 cm) with a similar number of males from each diet. To standardize nuptial gift investment, we only included virgin males (Karlsson, 1998) and individuals that were 4 days old or younger. Mated females were brought back to the lab to experience different humidity levels (see below).

### Humidity treatments

After mating, females were randomly assigned to one of two humidity treatments: dry (35% RH) or wet (65% RH). These treatments reflect natural humidity levels encountered across *P. rapae*’s range (Ryan et al., 2019), with wet being ideal, non-desiccating conditions (Meslin et al., 2017) whereas dry is a challenging, desiccating environment (Smith and Clarke, 1997). Females were kept for 48 h in individual cages (23×23×28 cm) within controlled greenhouses equipped with Inkbird humidity controllers and Dreyoo humidifiers, maintained in a temperature-controlled room at 23°C during the day and 20°C at night.

To control for water-saving strategies independent of nuptial gift use, virgin females were included in both humidity treatments. In *P. rapae*, sperm is transferred within the nuptial gift (spermatophore), making it impossible to obtain fertilized females without a nuptial gift. Consequently, comparisons between virgin and mated females reflect the combined effects of mating and nuptial gift receipt. We placed approximately 55 mated and 25 virgin females under each humidity condition. To standardize energy intake and ensure a uniform need to mobilize nuptial gift resources regardless of humidity effects, all females were hand-fed 5 µl of a 10% sucrose solution 24 h after entering the humidity treatment.

### Measuring the benefits of nuptial gifts on water balance and fitness

To assess the benefits of nuptial gifts for females experiencing dehydrating environments, we compared the water balance and survival of virgin and mated (with a nuptial gift) females when experiencing either a wet or a dry environment. We assessed changes in female water balance using haemolymph osmolality – the circulating fluid in invertebrates – and whole-body water content. Measuring haemolymph osmolality helps determine the concentration of solutes in the insect's circulatory fluid, which can increase during dehydration as water is lost from the body. However, osmolality can also be influenced by other factors, so it is important to consider those two complementary measurements together (Elnitsky et al., 2009). We predicted that dry conditions would increase osmolality and reduce water content, while nuptial gifts would buffer water loss by lowering osmolality and/or increasing water content. To collect haemolymph for osmolality measurements, females were first immobilized by placing them in a freezer (−20°C) for 3 min to facilitate handling. This duration is insufficient to induce chill coma and any minor transient effects would apply equally to all individuals, preserving the validity of comparisons among treatments. Then, a minimum 1 µl of haemolymph was extracted by piercing the thorax at the mesothorax–metathorax junction with a fine microcapillary tube. Samples were transferred to a microcentrifuge tube, diluted with 10 µl of stable saline solution (osmolality=285.8±1.7 mOsm kg^−1^) and stored frozen until analysis. Osmolality was measured using a Wescor Vapor Model 5600 osmometer. If more than 1 µl was collected, the sample was duplicated to assess repeatability, and values were adjusted for dilution. To assess measurement precision, we compared duplicate samples from 16 different individuals. Replicates differed on average by 2.1 mmol kg^−1^ (∼0.7% of the mean value), and a paired *t*-test showed no systematic difference between measurements (*P*=0.929), confirming the reliability of our osmolality estimates. Because haemolymph extraction was not always successful, valid osmolality measurements were obtained for a subset of individuals: dry conditions – 32 mated and 13 virgin females, wet conditions – 36 mated and 11 virgin females. Body water content (%) was calculated gravimetrically as: (wet mass−dry mass)/(wet mass)×100. Females were weighed before haemolymph collection to obtain the wet mass using a high precision scale (Sartorius Quintix65-1S), and again after being killed and dried at 50°C for 48 h to obtain dry mass. We included both measurements as they provide complementary information on the hydration state of individuals and are not directly correlated (Fig. S2).

Survival of mated and virgin females under the two different humidity conditions was monitored during the 48 h treatment period prior to sampling for water balance measurements. As females were already 3 days old at the onset of treatment, and their typical lifespan ranges from 5 days to 2 weeks, any mortality recorded within this window was used to assess whether dry conditions accelerated mortality between mated and virgin females.

### Evaluation of nuptial gift allocation under dry conditions and potential costs

To assess the potential costs of coping with dry conditions, we focused on both nuptial gift consumption and its allocation to reproduction and metabolism. We first quantified nuptial gift consumption over 48 h in both environments by comparing the dry mass of the remaining nuptial gift under dry versus wet conditions. While the initial mass of these nuptial gifts was not quantifiable, preliminary measurements indicated that nuptial gifts contain approximately 82% (3–4 mg) water immediately after mating (Table S1). We then assessed how nuptial gift-derived nutrients were used, specifically the allocation of leucine to either egg production or oxidation. To evaluate potential fecundity, we dissected mated females to remove their eggs and ovaries, dried the tissues at 50°C for 48 h, and recorded the dry mass.

At 48 h post-mating, we sampled breath isotopes in mated females using a CRDS analyser (G2121-i, Picarro, Santa Clara, CA, USA) to quantify CO_2_ production linked to gift-derived ^13^C-leucine (McCue et al., 2015). Male *P. rapae* were reared on an artificial diet enriched with ^13^C-leucine (as described above), resulting in nuptial gifts that were isotopically distinct from other female food sources (larval diet and beet sugar nectar, δ^13^C=−26.5‰; Fig. S1). Each female was placed in a 10 ml respirometry chamber for 10 min that was previously flushed with air previously deprived of CO_2_ and water (using a column containing soda lime and Drierite). Following the incubation, the chamber air was directed at 30 ml min^−1^ into the G2121-i CRDS stable carbon isotope analyser, with data recorded at 0.5 Hz using PICARRO software. CO_2_ and H_2_O measurement provided estimates of metabolic rate (*V̇*_CO_2__ in ml min^−1^) and respiratory water loss. Measurements were conducted in the afternoon within a 3 h window to limit diel variation, and the small chamber prevented flight. We chose to take measurements at 48 h based on prior work in *P. rapae* and similar species that indicated females start digesting the nuptial gift within the first hour after mating (Wedell and Cook, 1999) and that usage of nuptial gift-derived nutrients starts peaking on day 2 after mating in closely related species (Wiklund et al., 1993).

To compare how females under different %RH environments used nutrients (including nuptial gift-derived leucine), we dissected the abdomen of each female to separate eggs/ovaries from the fat body (a lipid-storage organ analogous to vertebrate liver and adipose tissue) and residual nuptial gift. Tissue samples were dried at 50°C for 48 h (VWR 1300U laboratory oven), homogenized and weighed. These samples were then loaded into tin capsules for δ^13^C measurement using a Picarro G2121-i stable isotope analyser. We obtained ^13^C concentrations of both control females (>20 per humidity treatment) and labelled females (>30 per humidity treatment) tissues, expressed in δ^13^CVPDB (Slater et al., 2001), and subsequently calculated the atom percentage (AP) and proportional ^13^C allocation to metabolism, eggs and somatic tissues (Slater et al., 2001). Because environmental conditions are known to influence natural stable isotope signatures, we first examined variation among control females (mated to non-labelled males) exposed to dry or wet conditions. In addition to tracing labelled leucine, we used the δ^13^C signatures of control females to assess endogenous nutrient allocation patterns. Because lipids are ^13^C-depleted relative to proteins and carbohydrates (DeNiro and Epstein, 1977), shifts in δ^13^C across tissues in control individuals provided insight into how water availability may influence macronutrient use. To account for this background variation in control females, we used atom percentage excess (APE), defined as APE=AP(label)−AP(control) (Welch et al., 2016), for subsequent analyses of leucine.

To analyse differences in relative allocation, AP values were converted into the mass of CO_2_ and ^13^CO_2_ in each pool (breath, eggs, fat body). For eggs and fat body, CO_2_ mass was calculated by multiplying the sample's CO_2_ concentration by the total dry mass of the pool (normalized by the sample mass), and the ^13^CO_2_ mass was derived by multiplying this value by the AP of ^13^C (divided by 100). For breath, we integrated the metabolic rate over 24 h to account for the timing of resource use from the nuptial gift, as females begin metabolizing it 1 day post-mating (Wiklund et al., 1993). Finally, to assess individual differences in nutrient allocation, we calculated the total ^13^C allocated to each pool and determined the relative allocation by dividing each pool's allocation by the total ^13^C allocation with the following calculation: (^13^C−leucine in pool)/total ^13^C. We hereafter refer to nuptial gift-derived leucine as the labelled leucine originating from the nuptial gift.

### Statistical analysis

#### Osmolality and body mass

We used linear mixed-effects models [lmer() from the lme4 package in R] to analyse osmolality and water content. The model included humidity treatment, female status (virgin or mated) and their interaction as fixed effects, with batch number as a random effect. Similarly, to compare egg production and the partial digestion of the nuptial gift in mated females, we modelled egg dry mass and residual nuptial gift dry mass as functions of humidity treatment and total dry body mass (with batch as a random effect). Model assumptions (normality and homoscedasticity of residuals) were verified through visual inspection of residuals and *Q*–*Q* plots.

#### Survival analysis

We first examined the survival rates of females under two different conditions: dry and wet. We performed a Mantel–Haenszel test (Mantel and Haenszel, 1959) to evaluate the association between mating status (virgin versus mated) and survival (survived versus died) across the two different treatments (dry and wet). The test was conducted using an exact method to account for the small number of deaths in some groups, with mortality being particularly low in the wet treatments (fewer than 5 individuals in both mated and virgin groups).

#### Isotopic measurements and metabolic rate

We first compared δ^13^C in eggs, fat body and breath of control females to assess how RH affected nutrient allocation in the absence of enrichment. We then compared isotopic signatures between females that received nuptial gifts from control (unlabelled) versus ^13^C-leucine-labelled males to test whether females allocated nuptial gift-derived leucine, an essential amino acid, to all three pools. We used linear mixed-effects models with AP as the response variable, and nuptial gift label, sample CO_2_ and pool dry mass (or total female dry mass for breath) as predictors, including batch as a random effect.

Second, to test the effect of RH on nuptial gift allocation, we analysed only females that received labelled nuptial gift. We used APE (Welch et al., 2016) as the response variable, with humidity treatment, pool mass and sample CO_2_ as predictors (again, using total female dry mass for breath).

Third, we assessed each individual's relative allocation of leucine by modelling the proportion of ^13^C in each pool relative to the total ^13^C. Finally, to test for trade-offs between allocation pathways, we modelled ^13^C allocation to one pool as a function of allocation to another, including an interaction with humidity treatment. Significance of fixed effects was assessed using Type II or III ANOVA (Anova function, car package).

Similar models were used to compare metabolic rate and evaporative water loss between dry and wet environments, with humidity treatment and female dry mass as covariates. Descriptive statistics are reported as means±s.e.m.

## RESULTS

### Benefits of nuptial gifts for water balance

**Fig. 1.**
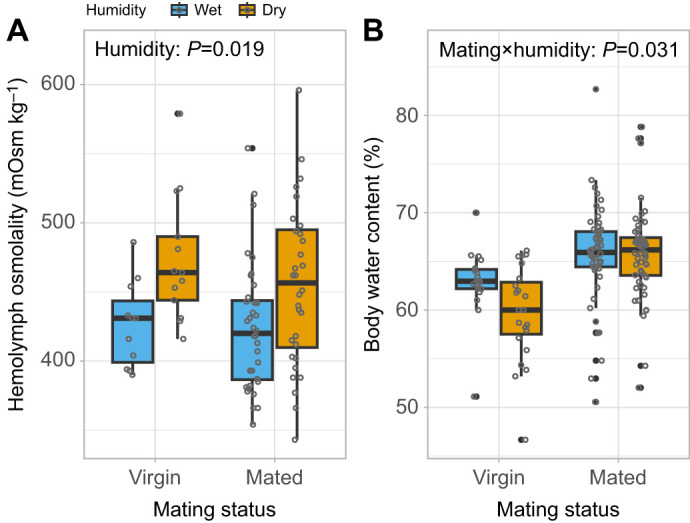
**Effect of mating status on water balance in *Pieris rapae* females exposed to either a wet (65% relative humidity, RH) or a dry (35% RH) environment.** Water balance was estimated using two complementary variables: (A) haemolymph osmolality and (B) body water content. Haemolymph osmolality was significantly affected by humidity treatment (*P*=0.019), and body water content showed a significant interaction between treatment and mating status (virgin versus mated) (*P*=0.031). Higher osmolality indicates dehydration, particularly when coupled with water loss. Boxplots show the median, interquartile range and data spread; individual data points are displayed as circles, with filled circles indicating statistical outliers (values outside the whiskers). Sample sizes: A, dry, mated *N*=32, virgin *N*=13; wet, mated *N*=36, virgin *N*=11; B, dry, mated *N*=56, virgin *N*=22; wet, mated *N*=54, virgin *N*=19.

Assessment of both haemolymph osmolality and body water content indicated that mated females were less dehydrated than virgin females when exposed to dry conditions. First, we found a significant main effect of humidity treatment on haemolymph osmolality, with females from the dry environment exhibiting higher osmolality compared with those from the wet environment (χ^2^=5.52, d.f.=1, *P*=0.019; osmolality in dry 460±8 mOsm kg^−1^, wet 425±6 mOsm kg^−1^; Fig. 1A). Second, the effect of humidity treatment on female total body water content was dependent on female mating status (χ^2^_humidity×mating_=4.65, d.f.=1, *P*=0.03; Fig. 1B); while virgin females showed reduced water content in the dry environment relative to the wet (mean±s.e.m. 59.7±1% in dry conditions; 62.8±0.8% in wet conditions), mated females maintained similar water levels across both environments (65.8±0.6% in dry; 65.7±0.7% in wet). Female mass, included as a covariate, also influenced water content (χ^2^_mass_=23.89, d.f.=1, *P*<0.001).

### Effect on fitness

Survival analysis results indicated that mated females had significantly higher odds of survival than virgin females across humidity environments (Mantel–Haenszel test, *P*=0.05; Fig. S4). Although the survival benefit of mating tended to be greater in dry conditions (a 10.7% increase in survival in dry versus 9.9% in wet conditions), this difference was not statistically significant (*P*=0.09). We also found no significant difference in female potential fecundity – measured as total dry mass of oocytes 48 h after mating – between mated females from dry and wet environments (*P*=0.46; Fig. S4).

### Water balance mechanisms

#### Respiratory water loss

**Fig. 2.**
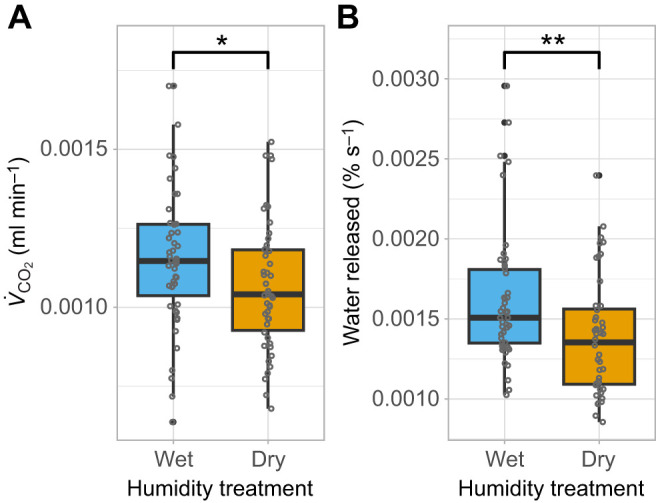
**Metabolic rate and water loss in *P.*** ***rapae*** **females that experienced either a wet (65% RH) or a dry (35% RH) environment in the 48 h following mating.** (A) Metabolic rate (*V̇*_CO_2__) and (B) water loss in breath. Boxplots show the median, interquartile range and data spread; individual data points are displayed as circles, with filled circles indicating statistical outliers (values outside the whiskers). Statistical significance between the two humidity groups was assessed using the Wilcoxon rank-sum test; *P<0.05, **P<0.01. Sample size, *N*=28 per treatment.

As expected, we found that respiratory water loss and metabolic rate were positively correlated, with higher *V̇*_CO_2__ values associated with greater respiratory water loss (*P*=0.006; Fig. S5). Importantly, females in the dry environment, compared with those in the wet environment, exhibited lower metabolic rate (χ^2^=4.12, d.f.=1, *P*=0.04; Fig. 2A) and significantly reduced water loss through the breath (χ^2^=9.31, d.f.=1, *P*=0.002; Fig. 2B), with female dry mass included as a significant covariate for metabolic rate (χ^2^=16.12, d.f.=1, *P*<0.001).

#### Nuptial gift consumption

We found no support for our hypothesis that females in dehydrating environments might accelerate consumption of nuptial gifts to obtain more water. Comparison of nuptial gift remains – their dry mass 48 h after mating – indicated that there was not a significant difference in consumption by females under dry versus wet environments (*P*=0.89; Fig. S3).

### Nutrient utilization and trade-offs

#### Female use of lipids

Comparison of δ^13^C in control females (mated to unlabelled males) indicated that females in the dry treatment had more-negative δ^13^C in eggs (*P*=0.03; Fig. S6) and less-negative δ^13^C in the fat body (*P*=0.05; Fig. S6) compared with females in the wet treatment. Given that lipids are naturally ^13^C depleted compared with proteins and carbohydrates (DeNiro and Epstein, 1977; Post et al., 2007), this suggests that females in dehydrating environments incorporated more lipids into eggs but less into storage. Comparison of δ^13^C_breath_ among treatments revealed no evidence of altered lipid catabolism under dehydrating conditions (*P*=0.76).

#### Female use and allocation of nuptial gift-derived leucine

**Fig. 3.**
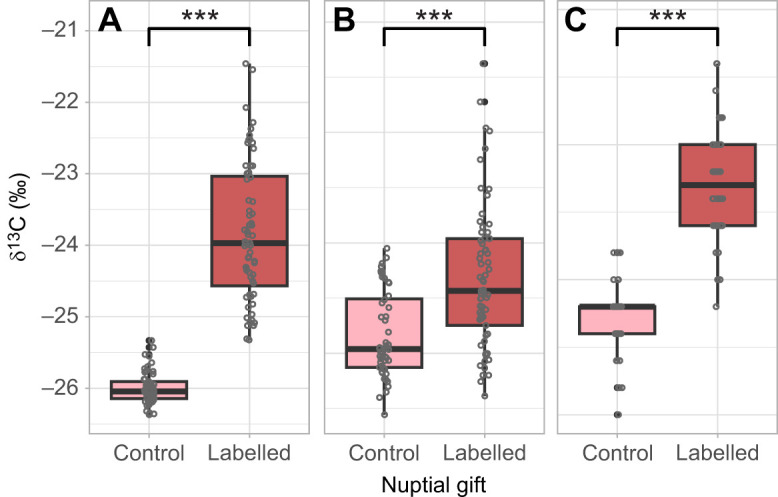
**δ^13^C values in different body parts of *P. rapae* females that received either a control or a ^13^C-leucine labelled nuptial gift.** δ^13^C values are shown in eggs (A), fat body (B) and breath (C) to demonstrate allocation of nuptial gift-derived leucine to reproduction, storage and metabolism. Less-negative δ^13^C values indicate higher tracer content. Boxplots show the median, interquartile range and data spread; individual data points are displayed as circles, with filled circles indicating statistical outliers (values outside the whiskers). Sample sizes: labelled nuptial gift *N*=64; control nuptial gift *N*=48. ***P<0.005.

Comparison of δ^13^C in females receiving a control versus a ^13^C-leucine labelled nuptial gift revealed that females deposited this essential amino acid both into eggs (χ^2^=262, d.f.=1, *P*<0.001; Fig. 3A) and into storage (χ^2^=23.2, d.f.=1, *P*<0.001; Fig. 3B). Also, breath analysis further revealed significant differences in δ^13^C between the two female groups (χ^2^=217, d.f.=1, *P*<0.001; Fig. 3C), indicating that females used nuptial gift-derived leucine as a metabolic fuel.

**Fig. 4.**
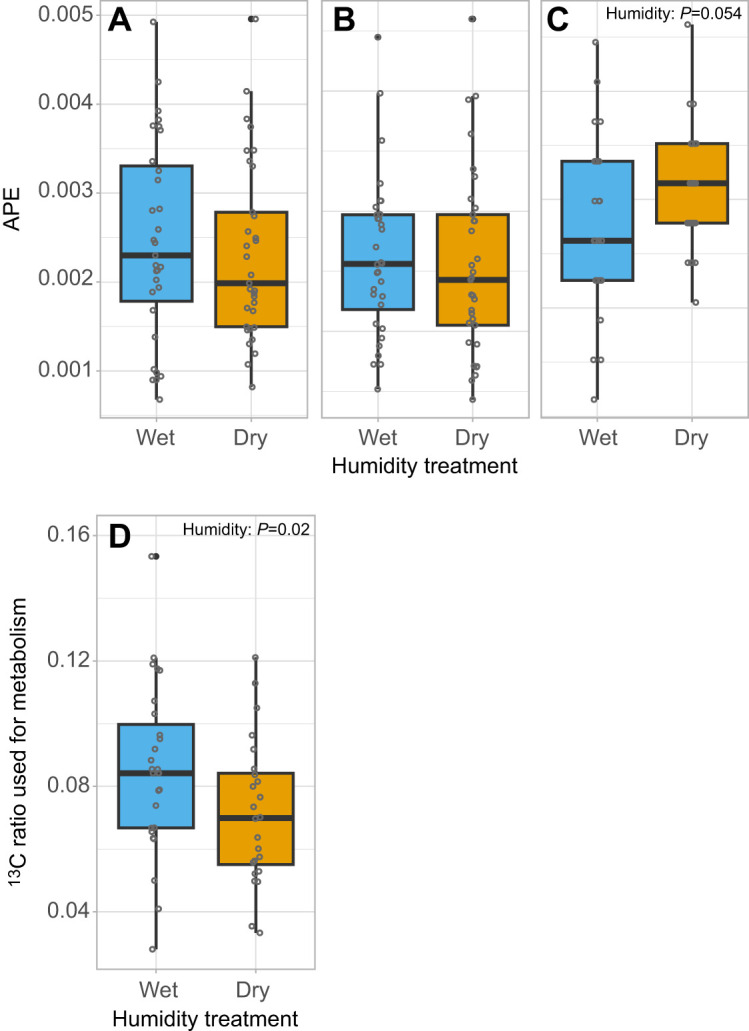
**Allocation of ^13^C-leucine labelled nuptial gift in *P. rapae* females that experienced either a wet (65% RH) or a dry (35% RH) environment after mating.** ^13^C enrichment is shown as atom percentage excess (APE, in ^13^C) in eggs (A), fat body (B) and breath (C) to demonstrate different allocation of nuptial gift-derived leucine to reproduction, storage and metabolism under two humidity conditions. The ratio of ^13^C-leucine used as fuel for metabolism in 24 h over the total female usage of ^13^C-leucine is also shown (D). Boxplots show the median, interquartile range and data spread; individual data points are displayed as circles, with filled circles indicating statistical outliers (values outside the whiskers). Sample sizes: A and B, dry *N*=33, wet *N*=31; C and D, *N*=28 per treatment.

RH did not significantly affect the absolute amount of nuptial gift-derived leucine allocated to eggs (*P*=0.25; Fig. 4A) or stored in the fat body (*P*=0.45; Fig. 4B), but did lead to a marginally significant effect on leucine catabolism, with females in the dry treatment increasing leucine oxidation compared with those in the wet treatment (breath APE: estimate=0.0011±0.0005, *P*=0.054; Fig. 4C). At the same time, when comparing the relative allocation of total recovered nuptial gift-derived leucine, females in dry environments allocated a smaller share for catabolism (calculated as the ratio of ^13^C_breath×24h_ /^13^C_total_ _recovered_) than those in wet environments (χ^2^=5.37, d.f.=1, *P*=0.02; Fig. 4D); this test accounted for the significant effects of female dry mass (χ^2^=22.98, d.f.=1, *P*<0.001). Finally, no significant differences were observed in the relative allocation of leucine to eggs (*P*=0.51) or to the fat body (*P*=0.77) between humidity treatments.

Although we found evidence that the allocation of nuptial gift-derived leucine to metabolism could directly reduce its availability for other functions, this was independent of humidity treatment. Overall, females that oxidized a greater proportion of leucine also allocated more to egg production (χ^2^=4.19, d.f.=1, *P*=0.04), with no significant effect of humidity (estimate=0.97). In contrast, leucine oxidation was negatively associated with allocation to the fat body (χ^2^=17.34, d.f.=1, *P*<0.001), again independent of humidity treatment (estimate=−1.97).

## DISCUSSION

Across taxa, maintaining effective water balance is crucial in water-limited environments. However, the costs and trade-offs associated with increasing water intake or reducing water loss remain largely underexplored, limiting our ability to fully understand and predict how organisms respond to increased drought and temperature extremes (McCluney, 2017; Sinclair et al., 2024). In this study, we addressed this issue by investigating how water balance, enhanced by the nuptial gift received during mating, affects female nutrient use and fecundity in *P. rapae* butterflies experiencing dehydrating environments.

### Impact of the nuptial gift on female hydric state and fitness

Although primarily seen as a form of paternal investment (Vahed, 1998), nuptial gifts can also play a crucial role in enhancing stress tolerance by supporting female water balance, especially in species experiencing variable conditions (Gordo et al., 2010). Yet, evidence for the potential hydration benefits of nuptial gifts has thus far been primarily indirect, relying on studies examining female mating frequency in relation to water availability or comparing offspring production under hydrated versus dehydrated conditions (Edvardsson, 2007; Ivy et al., 1999; Ursprung et al., 2009). Our study provides the first direct physiological evidence that male nuptial gifts help females maintain water balance, demonstrating that a single gift increased female body water content by over 5% under dry conditions.

However, body water content alone does not fully capture hydration status. To better assess the physiological effects of mating, we also measured haemolymph osmolality, which can rise not only from water loss but also from the absorption of solutes such as amino acid or salts during nutrient mobilization (Chown and Nicolson, 2004). We observed pronounced within-treatment variation in both osmolality (interquartile range, IQR: 51.5 mOsm kg^−1^ in wet and 76 mOsm kg^−1^ in dry conditions) and body water content (IQR: 4.5% in wet and 5.8% in dry conditions), a pattern consistent with substantial physiological heterogeneity commonly reported within insect species and likely arising from a combination of genetic diversity and strong condition dependence (Benoit et al., 2010; Dahake et al., 2024; Sinclair et al., 2024). Despite this natural variation, clear treatment-level patterns emerged: under dry conditions, osmolality increased in both mated and virgin females, but only virgins exhibited a concurrent decrease in body water content, indicating true dehydration. In contrast, mated females retained more water despite the osmolality increase, suggesting that elevated solute levels – possibly from nutrient uptake via the nuptial gift or active osmoregulatory processes – rather than water loss, contributed to the elevated osmolality (Elnitsky et al., 2009). This supports the idea that the nuptial gift buffered females against dehydration and helped them maintain cellular hydration under dry conditions.

Unexpectedly, nuptial gifts offered no measurable benefit for female survival or fecundity in dehydrating environments. Although our experimental treatments were impactful – dry conditions increased mortality and mated females showed better overall survival, probably due to the nuptial gift's nutritional benefits (Boggs, 1995) – the physiological hydric benefits of the gifts did not translate into a clear fitness advantage. This may partly reflect the temporal scale of our measurements, as we focused on potential fecundity and early survival during the 48 h following mating, even though females were already several days old and thus well within the time frame where harsh conditions could meaningfully influence lifetime fitness. Nonetheless, fitness effects may become evident later, during egg maturation or through differences in egg water content and subsequent hatching success. Altogether, these results highlight the value of physiological measures, which can reveal early and subtle changes in female condition before they become evident in fitness outcomes.

### Water balance mechanisms and nuptial gift use

We found that a single *P. rapae* nuptial gift, containing over 80% water by wet mass, could provide approximately 3–4 mg of preformed water. This amount closely matches the body water gain observed in mated females under dry conditions, suggesting that the direct intake of the nuptial gift's water could account for a significant portion of the observed hydration benefit. However, the mechanism by which *P. rapae* utilize the gift's water appeared to be complex, as females did not alter the consumption rate of it in dry conditions. This suggests that females do not fully control the rate of water (and nutrient) uptake; instead, this process may be largely influenced by the male ejaculate composition (Plakke et al., 2015).

The observed hydration gain, despite the lack of increased water intake via consumption, suggests that complementary water balance mechanisms, including water loss reduction and metabolic water production, may explain how females benefit from the gift. Indeed, females in dry conditions significantly decreased metabolic rate, which thus reduced their respiratory water losses by almost 15%. This is a common physiological adaptation observed across diverse insect taxa for coping with water scarcity (Chown and Davis, 2003; Marron et al., 2003; Woods and Smith, 2010). Interestingly, we also found some indication that the catabolism of nutrients derived from the nuptial gift could contribute to water balance in *P. rapae* females. Specifically, we observed a trend toward increased catabolism of nuptial gift-derived leucine under dry conditions. Protein (or amino acid) catabolism is an important mechanism for water generation in some vertebrate species (Brusch et al., 2018; Gerson and Guglielmo, 2011; Rogers and Gerson, 2024). Although uricotelic insects excrete nitrogen with minimal water loss – suggesting amino acid catabolism could efficiently generate internal water – evidence for this strategy is notably absent within insect taxa. Instead, studies have shown that insects employ alternative strategies, favouring lipid metabolism (Loveridge and Bursell, 1975) or relying on carbohydrate catabolism – particularly in desert-adapted species (Kalra and Gefen, 2012; Marron et al., 2003).

Interestingly, although there was a trend for a greater proportion of expired carbon in dry females to be derived from nuptial gift-derived leucine, a smaller share of their total recovered nuptial gift-derived leucine was allocated towards catabolism compared with those in wet environments. This shift was at least partly driven by the observed reduction in metabolic rate among females in dry conditions. Indeed, organisms across diverse taxa often reduce their metabolic overhead during periods of environmental stress. We see this in insects entering diapause (Denlinger, 2002), birds and mammals undergoing torpor (Ruf and Geiser, 2015) and even desert-dwelling amphibians decreasing metabolic activity to conserve both water and energy during dormancy (Withers, 1993). Our results, therefore, indicate an integration of water-saving mechanisms with broader nutrient conservation strategies when facing challenging environmental conditions.

### Shifts in nutrient allocation and trade-offs

Our study revealed strategic reallocation and trade-offs in the utilization of both lipids and nuptial gift-derived leucine by females in dehydrating environments. Although we found no evidence of increased lipid catabolism, females in dry environments increased lipid deposition into eggs while simultaneously reducing lipid storage in the fat body. This pattern is consistent with reproductive trade-offs commonly observed in insects, where limited resources are prioritized for immediate reproductive output at the expense of future reproduction (Ellers, 1996; Xu et al., 2024). However, increased lipid content does not necessarily improve egg quality, as some studies have shown that eggs richer in lipids have lower protein content, which can negatively affect hatching success (Geister et al., 2008). Because lipids may originate from both the larval diet and the nuptial gift – and did not appear to contribute to metabolic water production – the observed lipid shifts likely reflect broader changes in how females manage internal resources under environmental stress. One possibility is that by allocating more lipids to eggs, females conserve protein and glycogen for their own somatic use, as these substrates generate more metabolic water during catabolism (Jenni and Jenni-Eiermann, 1998) – a potentially advantageous strategy in dry environments.

Females broadly allocated leucine across eggs, storage and metabolic fuel. Importantly, they exhibited a clear allocation trade-off, prioritizing the nuptial gift-derived essential amino acid for immediate metabolic needs and reproduction over long-term storage. For essential amino acids, which cannot be synthesized, their immediate use for vital processes not only provides direct benefits but also avoids costs associated with storing nutrients: energy for synthesis, maintenance and potentially breakdown later (Iromini et al., 2023). Intriguingly, despite our prediction that water stress would alter nuptial gift resource allocation, leucine allocation trade-offs remained consistent across dry and wet environments. Our results therefore indicate that, for this essential amino acid, the female's strategic prioritization of immediate reproduction and metabolic function overrides environmental water availability.

In summary, our findings indicate that nuptial gifts aid females in coping with dehydration by directly supplying water and by enabling flexible nutrient allocation decisions that optimize fitness under stress. Thus, the primary value of nuptial gifts in dehydrating environments may not be enhancing short-term fitness (survival or fecundity) but supporting the physiological adjustments necessary for reproduction to continue during stressful conditions. This crucial flexibility – evident in altered lipid allocation, selective nutrient catabolism and changes in metabolic rate – likely underpins the ecological success of *P. rapae* in environments with variable water availability and illustrates a powerful intersection between mating interactions and stress physiology in shaping fitness outcomes under climate change.

### Implications and future perspectives

Our study shows that water limitation reshapes nutrient allocation, with direct consequences for female performance and reproduction. Because water balance interacts closely with nutrient and metabolic processes, understanding how these physiological demands intersect is critical as global aridity intensifies. Future work that elucidates the mechanisms and strategies organisms use to integrate water and nutrient constraints will be key to predicting responses to increasingly variable and resource-limited environments.

These findings highlight the adaptive significance of nuptial gifts in water-limited environments and raise questions that extend across taxa. If gifts contribute to female hydration, their production may impose additional costs on males under hydric stress. This challenges the idea that water in nuptial gifts serves primarily as a manipulative tool to delay female remating (Oberhauser, 1989; Sugawara, 1979) and instead suggests a potentially mutualistic role. Large gifts may be favoured for their dual function: extending the refractory period while providing water and other nutrients (Vahed, 1998). Although most research on water provisioning has focused on insects, resource-based courtship feeding also occurs in other taxa, such as birds, where males provide food items to females as part of mate assessment or copulation exchange (Tryjanowski and Hromada, 2005). Exploring these dynamics across taxa will generate generalizable insights into how organisms integrate hydration and nutrient constraints, ultimately shaping reproductive strategies, species resilience and community responses in drought-prone and variable environments.

## Supplementary Material

10.1242/jexbio.251506_sup1 Supplementary information
