# Supplementary material for: Shifts in nutrient allocation in a gift-giving butterfly: a hidden consequence of water balance?
Source: J Exp Biol. 2026 Jan 23;229(2):jeb251506. doi: 10.1242/jeb.251506 (PMC12863298; doi:10.1242/jeb.251506)
Supplement: Supplementary information [file jexbio-229-251506-s1.pdf]

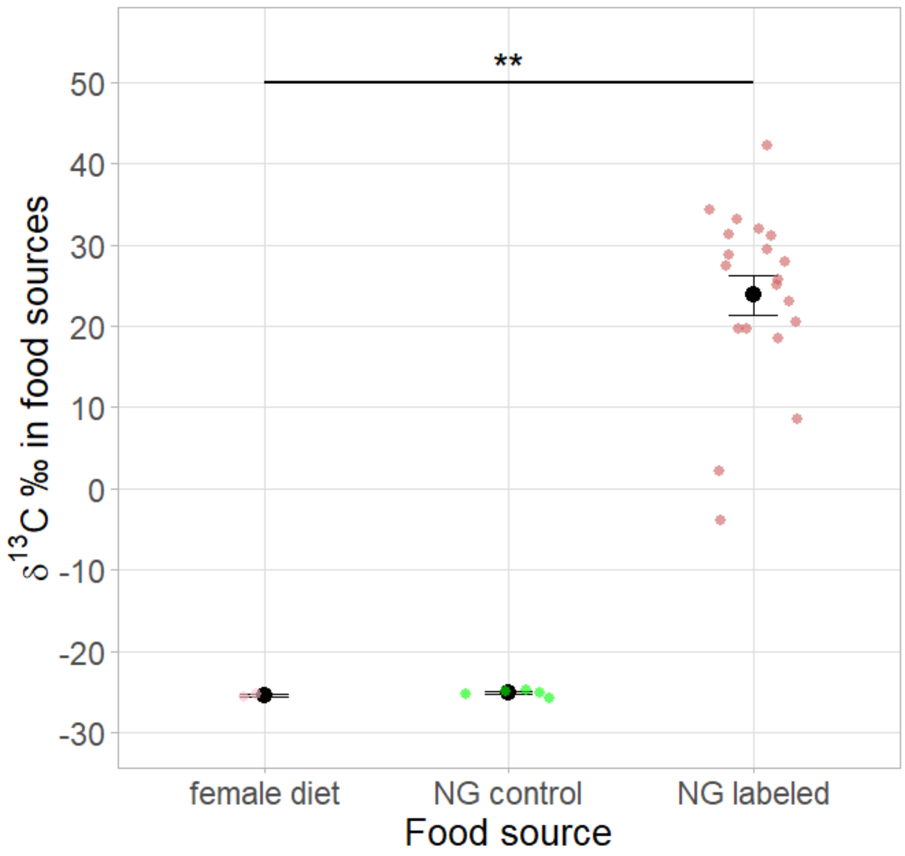


**Fig. S1. δ$^{13}$C signature of control and labelled nuptial gifts, compared to the female larvae diet.** Plot showing the δ$^{13}$C values across food sources available to *P. rapae* females. Nuptial gifts obtained from labelled males had significantly higher δ$^{13}$C signatures than both the female larval diet and the nuptial gifts obtained from control males. **$p < 0.01$

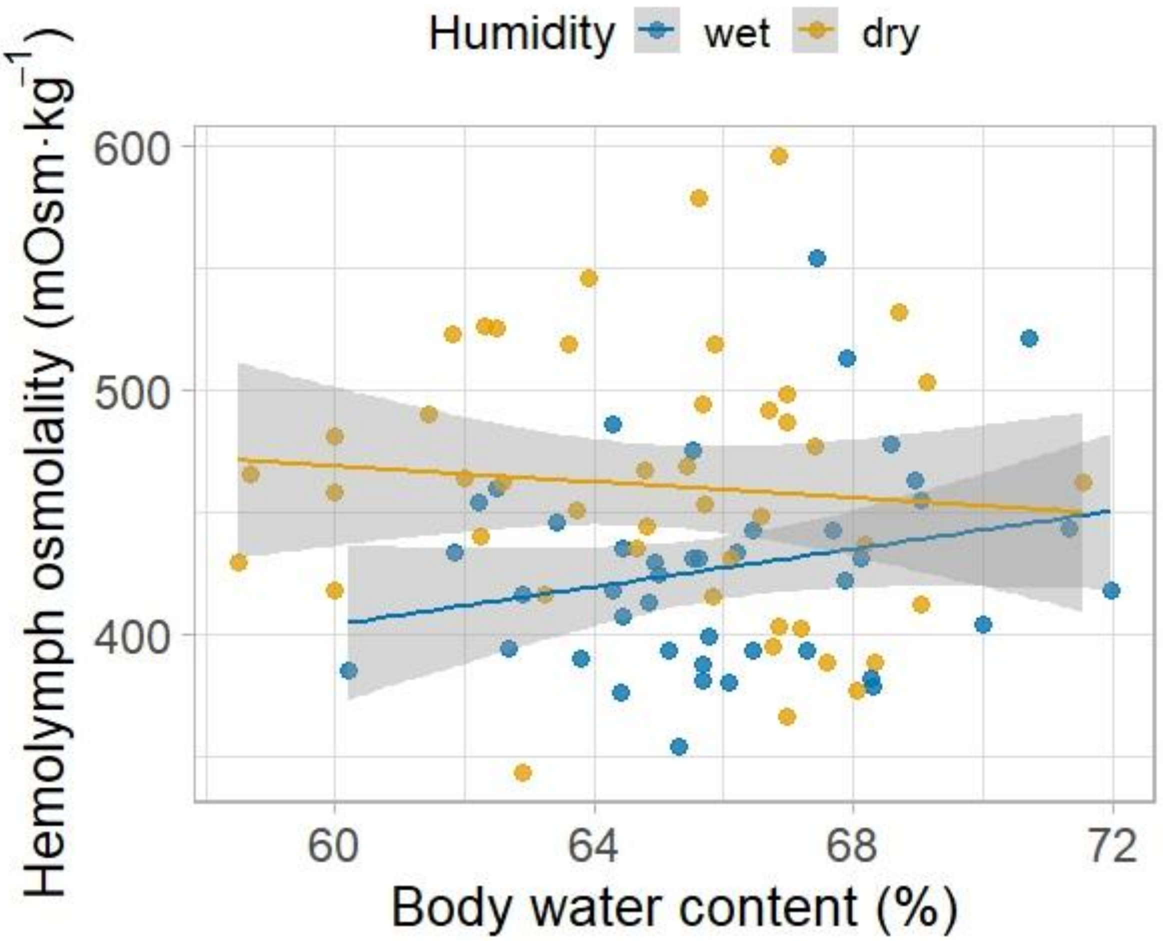


**Fig. S2. Relationship between individual body water content and haemolymph osmolality.** Relationship between body water content and hemolymph osmolality in *Pieris rapae* females. Each point represents an individual, with colours indicating humidity treatment (blue = wet, orange = dry). Lines show linear regressions with 95% confidence intervals. No significant relationship was detected between body water content and hemolymph osmolality (linear regression: $R^2 < 0.05$, $p = 0.76$).

Journal of Experimental Biology • Supplementary information

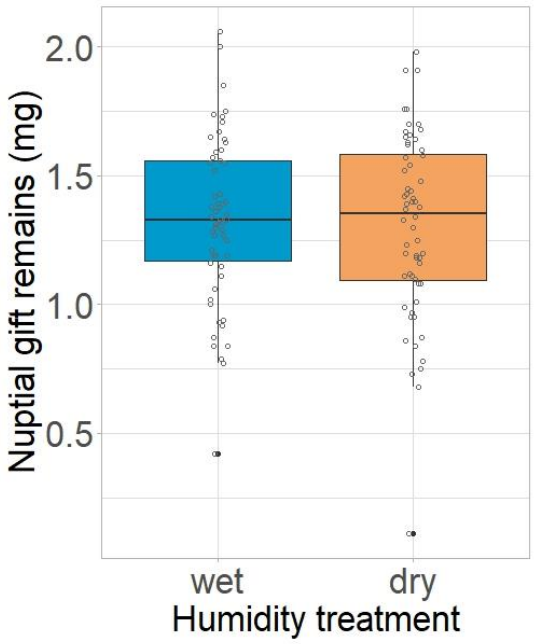


**Fig. S3. Nuptial gift consumption in dry and wet environments** Boxplot showing the distribution of the dry masses of nuptial gift remains from *P. rapae* females that experienced either a wet (blue, 65% RH) or a dry (orange, 35% RH) environment for 48 hours after mating. This measure was used to assess nuptial gift consumption under different humidity conditions. Boxplots show the median, interquartile range, and data spread; individual data points are shown as crosses. No significant difference was observed between treatments.

Journal of Experimental Biology • Supplementary information

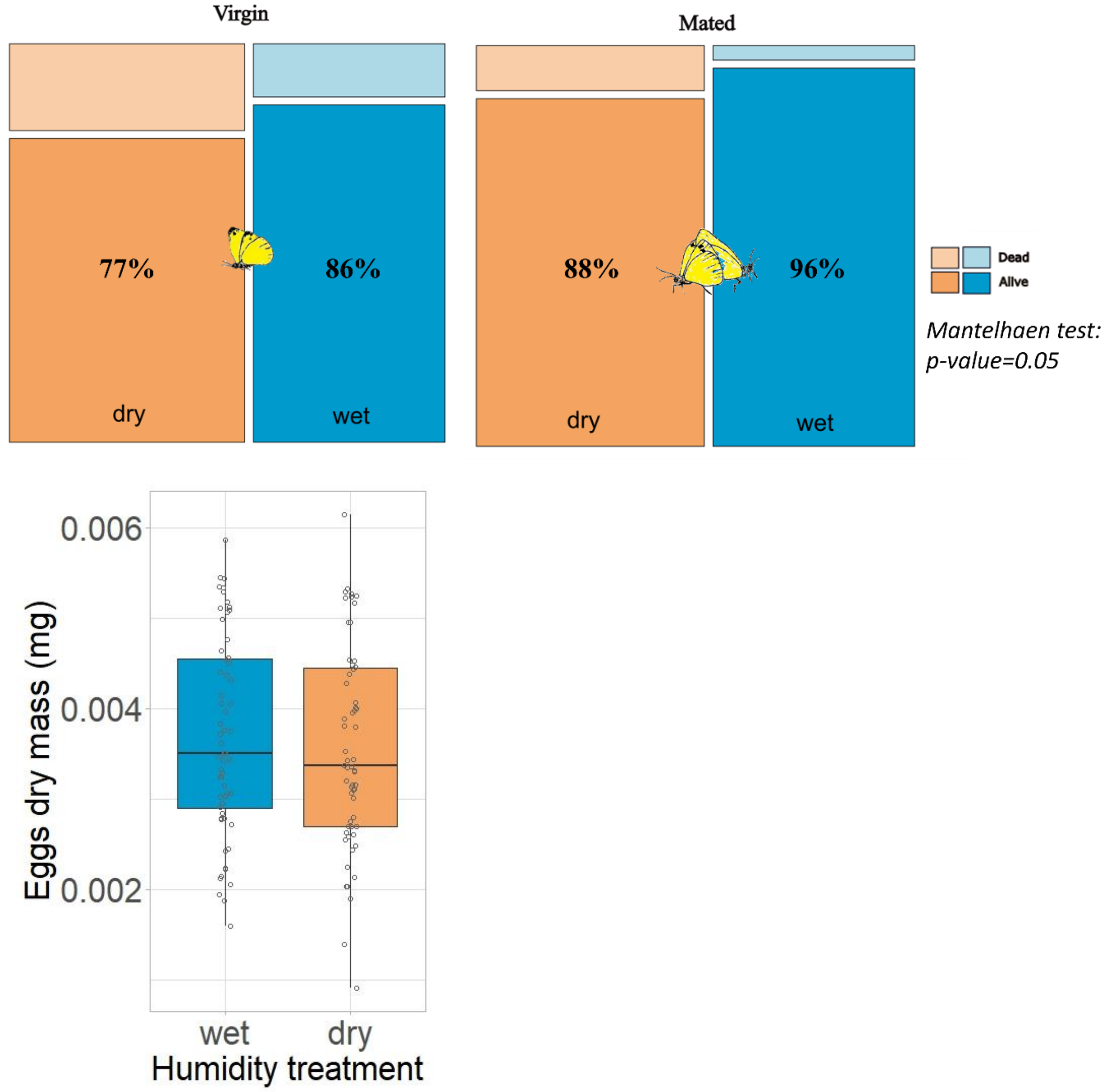


**Fig. S4. Fitness consequences of wet versus dry environments (A)** Survival probability of *P. rapae* females during the 48-hour experimental period in either a wet (blue, 65% RH) or dry (orange, 35% RH) environment. The left panel shows virgin females; the right panel shows mated females. Survival was significantly lower in the dry environment overall (Mantel-Haenszel test, $p = 0.05$) **(B)** Potential fecundity of *P. rapae* mated females after 48 hours in either a wet or dry environment, measured as the dry mass of eggs and ovaries. No significant difference was observed between treatments.

Journal of Experimental Biology • Supplementary information

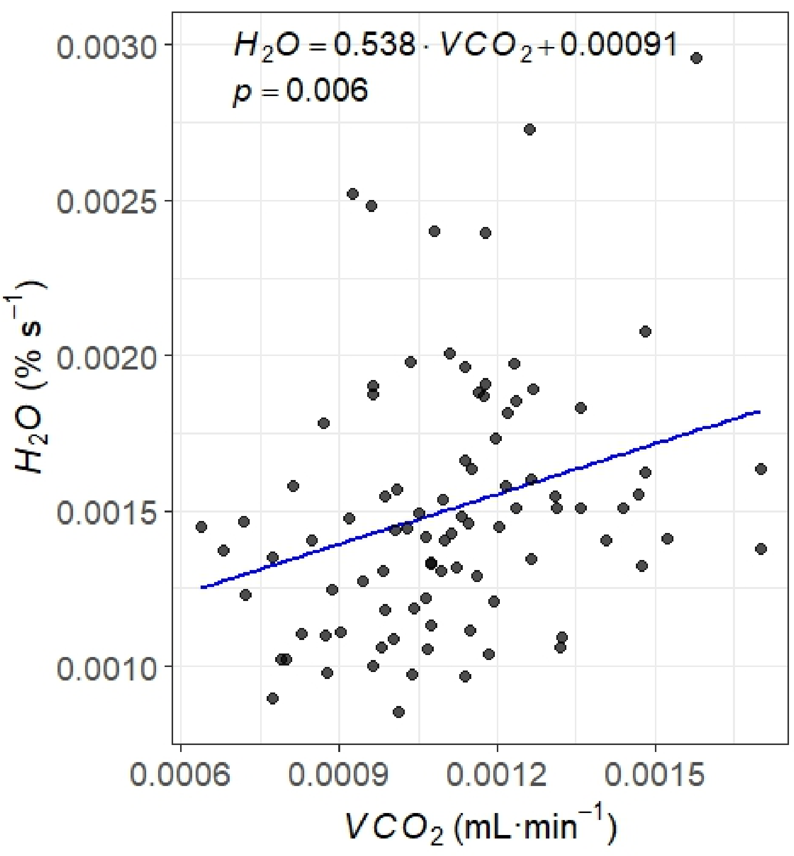


**Fig. S5. Relationship between metabolic rate and water loss.** Relationship between $V_{CO2}$ (mL/min) and water loss (% per second) during the 10-minute respirometry trial in *P. rapae* mated females. The solid line represents the linear fit. A Spearman correlation was used to assess the strength and direction of the relationship ($\rho = 0.34$, $p = 0.00091$).

Journal of Experimental Biology • Supplementary information

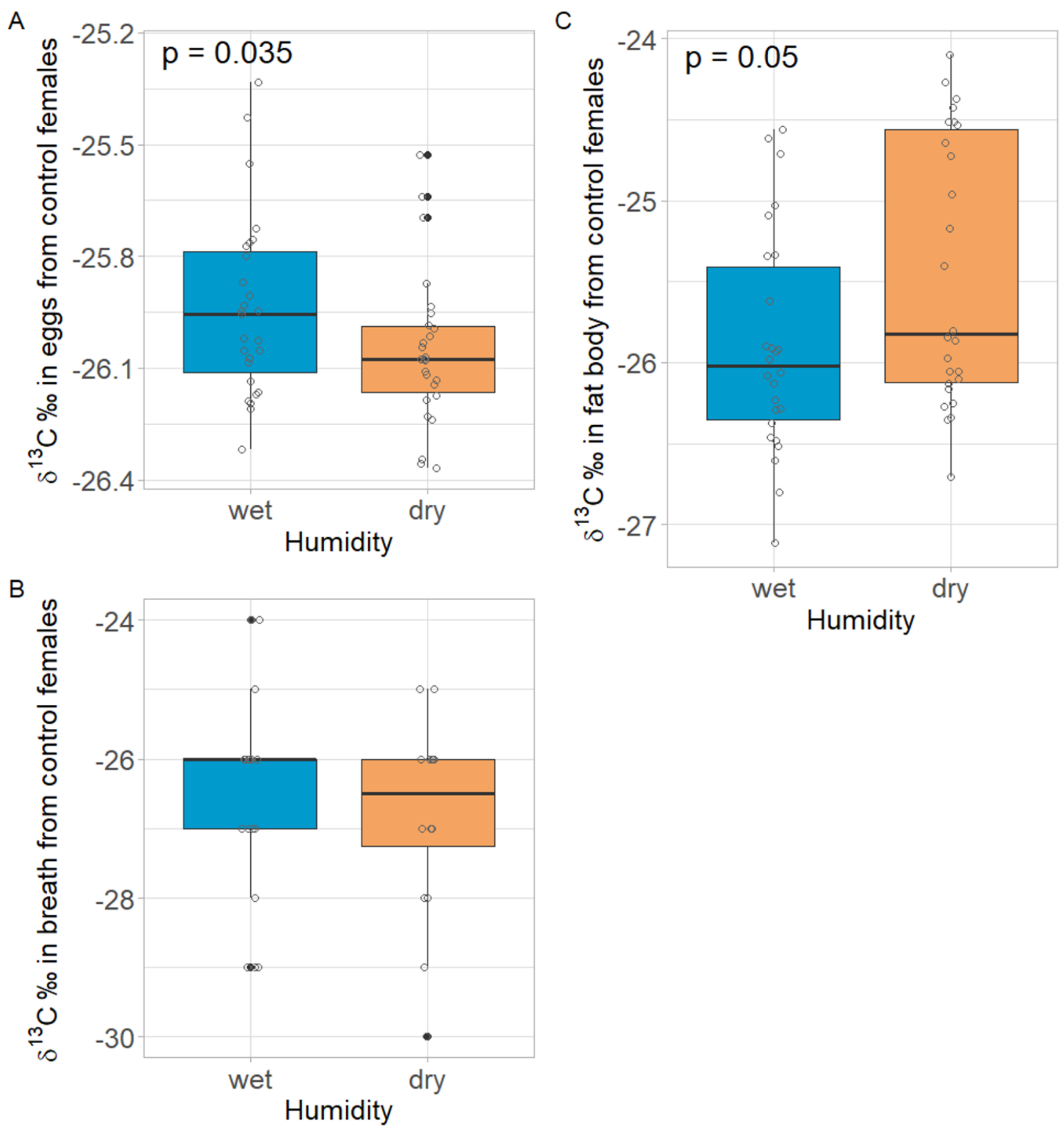


**Fig. S6. $\delta^{13}C$ signature of eggs, fat body and breath from control females experiencing different environment.** Isotopic signatures ($\delta^{13}C$) of control females mated with unlabelled males, reflecting the relative allocation of lipids versus proteins and carbohydrates across three physiological pools: (A) eggs (reproduction), (B) breath $CO_2$ (metabolism) and (C) fat body (storage). Because lipids are naturally $^{13}C$-depleted compared to proteins and carbohydrates, more negative $\delta^{13}C$ values indicate greater lipid contribution. Under dehydrating conditions, females exhibited more $^{13}C$-depleted eggs and less $^{13}C$-depleted fat bodies, suggesting increased lipid allocation to reproduction and reduced lipid storage.

Journal of Experimental Biology • Supplementary information

**Table S1. Water content of complete nuptial gifts (N=25):** Mean (± SE) dry mass, wet mass, and water content of 25 spermatophores from first male copulations in *Pieris rapae*. Spermatophores were removed from females less than 1 hour after copulation, before females began consuming them. Mated females were dissected to remove the bursa copulatrix, from which the spermatophore was extracted, weighed immediately to obtain wet mass, then dried at 50 °C for 48 h before recording dry mass.

| **Wet mass (mg)** | **Dry mass (mg)** | **Water content (%)** |
|---|---|---|
| **5.05 ± 0.29** | **0.84 ± 0.06** | **82.85** |
